# Detection of leukocoria using a soft fusion of expert classifiers under non-clinical settings

**DOI:** 10.1186/1471-2415-14-110

**Published:** 2014-09-09

**Authors:** Pablo Rivas-Perea, Erich Baker, Greg Hamerly, Bryan F Shaw

**Affiliations:** Department of Computer Science, Baylor University, One Bear Place #97356, Waco, TX 76798-7356 USA; Department of Chemistry & Biochemistry, Baylor University, One Bear Place #97348, Waco, TX 76798-7348 USA

**Keywords:** Leukocoria, Retinoblastoma, Fuzzy logic, Soft computing, Discrete cosine transform, Karhunen-Loeve transform

## Abstract

**Background:**

Leukocoria is defined as a white reflection and its manifestation is symptomatic of several ocular pathologies, including retinoblastoma (Rb). Early detection of recurrent leukocoria is critical for improved patient outcomes and can be accomplished via the examination of recreational photography. To date, there exists a paucity of methods to automate leukocoria detection within such a dataset.

**Methods:**

This research explores a novel classification scheme that uses fuzzy logic theory to combine a number of classifiers that are experts in performing multichannel detection of leukocoria from recreational photography. The proposed scheme extracts features aided by the discrete cosine transform and the Karhunen-Loeve transformation.

**Results:**

The soft fusion of classifiers is significantly better than other methods of combining classifiers with *p* = 1.12 × 10^-5^. The proposed methodology performs at a 92% accuracy rate, with an 89% true positive rate, and an 11% false positive rate. Furthermore, the results produced by our methodology exhibit the lowest average variance.

**Conclusions:**

The proposed methodology overcomes non-ideal conditions of image acquisition, presenting a competent approach for the detection of leukocoria. Results suggest that recreational photography can be used in combination with the fusion of individual experts in multichannel classification and preprocessing tools such as the discrete cosine transform and the Karhunen-Loeve transformation.

**Electronic supplementary material:**

The online version of this article (doi:10.1186/1471-2415-14-110) contains supplementary material, which is available to authorized users.

## Background

Leukocoria is an abnormal pupillary light reflex that is characterized by a persistent ‘white-eye’ phenomenon during visible light photography. It is often the primary observable diagnostic symptom for a range of catastrophic ocular disorders. In addition, leukocoria is a prevailing symptom of congenital cataracts, vitreoretinal disorders and malformations, retinopathy of prematurity, trauma-associated diseases, Coats’ disease, ocular toxocariasis, Norrie disease, ciliary melanoma, retrolental fibroplasia, and retinal hamartomas [1, 2], see [3] for a review. In children under the age of 5, however, the predominant cause of leukocoria is Rb [4, 5].

In the case of Rb, tumors in the eye can act as diffuse reflectors of visible light [6–9]. Consequently, leukocoria associated with Rb is a progressive symptom that occurs more frequently, during recreational photography, as the size and number of tumors increase [10]. The fact that it occurs in recreational photography opens the door to investigate a way to perform an automatic assessment of visual dysfunction [11]. Leukocoria is optically distinct from specular reflections of the cornea and can be detected with a low resolution digital camera, a camera phone equipped with or without a flash, or with a digital video recorder. In clinical settings, the "red reflex" test is adequate for the identification of tumor reflections when administered by trained clinicians, but may suffer from a high degree of false negatives when conducted under a wide range of conditions [12, 13]. This ineffectiveness of the "red-reflex" test is especially problematic in developing nations where there is a limited supply of properly trained specialists in ophthalmology or pediatrics. Even in developed nations, recent studies suggest that clinicians are either improperly trained for leukocoric screening, or do not perform the test [14]. Indeed, parents or relatives are generally the first individuals to detect leukocoria in a child, and their observation often initiates diagnosis [1, 4, 15–17]. For example, in a study of 1632 patients with Rb, the eventual diagnosis in ∼80% of cases was initiated by a relative who observed leukocoria in a photograph [4].

The consequences of a false negative finding can be profound, as the case of Rb illustrates. While it only comprises 3-4% of pediatric cancer, the incidence of Rb is high enough (i.e., ∼ 1-2:30,000 live births) to mandate universal screening [4, 13]. The median age of diagnosis is 24 months for unilateral disease and 9–12 months for bilateral disease [18, 19]. When detected early, Rb is curable, either by enucleation of the eye, or the use of ocular salvage treatments with chemotherapy and focal treatments or radiation therapy [20, 21]. Delays in diagnosis lead to increased rates of vision loss, need for therapy intensification (with its associated life-time toxicity) and death, particularly for children who live in resource-poor settings [7]. Compressing diagnostic time frames rely, in part, on improved methods for detecting intraocular tumors or their leukocoric presentation.

The autonomous and semi-autonomous analysis of diagnostic medical images, such as those mediated by computational biology and machine learning, are routinely used for the unsupervised and supervised prediction and prognosis of numerous pathologies and pathology outcomes, but have had limited application in areas of detection and diagnosis [22, 23]. In applications where machine learning has been applied to the discernment of disease based on image data (analogous to the observable detection of leukocoria in digital photographs), there has been significant success. These previous studies have employed a variety of soft computing techniques: support vector machines (SVMs), Bayesian statistical approaches and neural networks have been used to assist in the detection of breast cancer in mammograms [24], prostate cancer [25], lung cancer [26] and cervical cancers [27]. Of particular importance has been the successful use of neural networks for the detection of skin cancers, such as melanoma, where non-histological photographic digital images serve as the medium [28–31]. In each of these scenarios, however, studies have been applied to controlled environments where skilled technicians intentionally seek to classify disease states.

**Figure 1:**
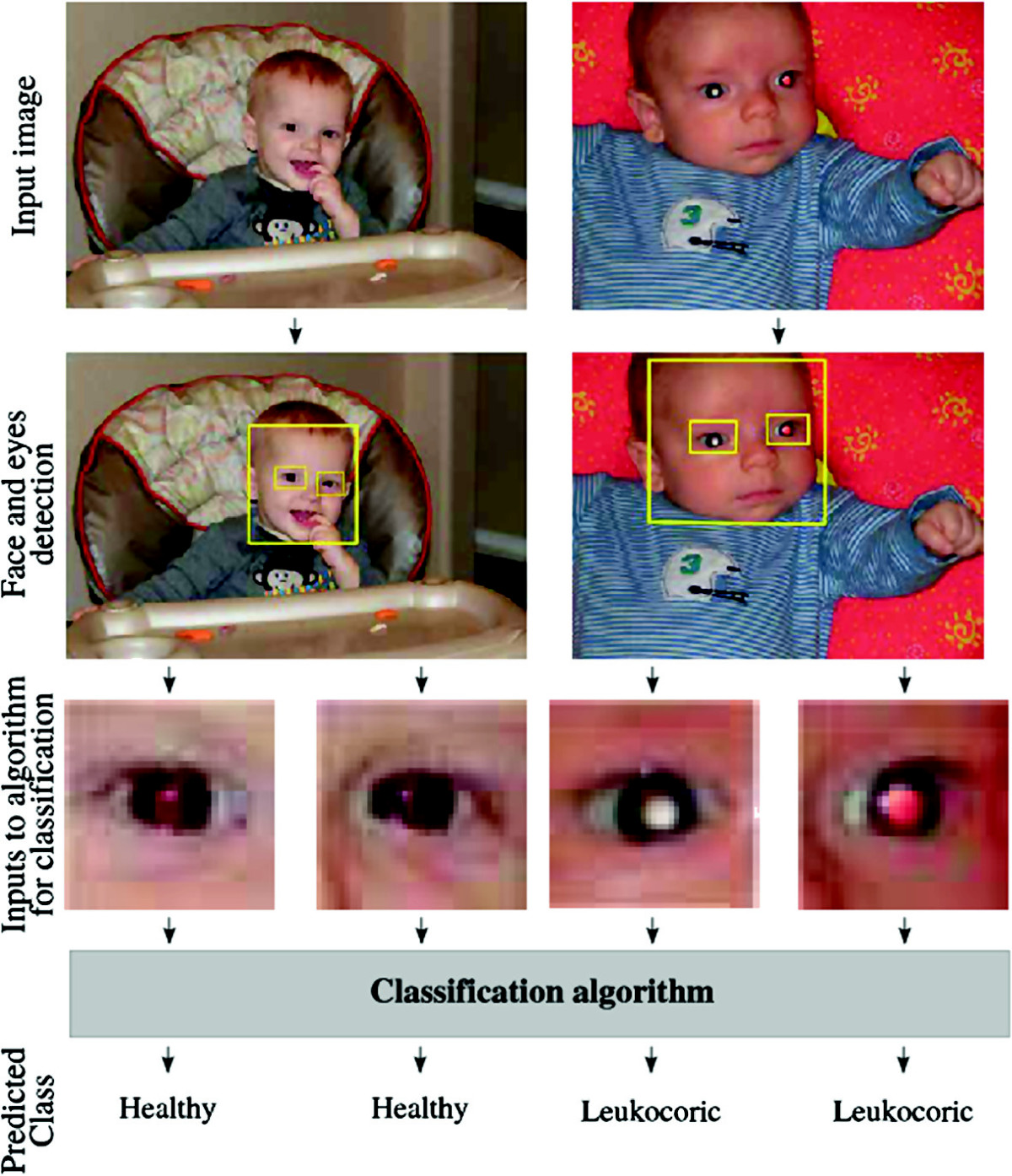
**Process of classification of two input images.**

In spite of the apparent symptomology and recent successes in categorization [10], the automated or semi-automated detection of leukocoria remains a naive process. Therefore, this paper proposes a classification algorithm that detects a leukocoric eye using images (see Figure 1) processed to automatically detect faces and the position of the eyes [32], regions of interest, i.e., both eyes, and, finally, an individual class for each eye using a soft fusion of multiple classifiers to produce optimal results. The essential property of soft fusion of classifiers is the use of fuzzy integrals as a similarity measure [33, 34]. While still a very active area of research [35, 36], the fusion of multiple classifiers based on support vector machines, neural networks, and discriminant analysis has had success, such as the classification of bacteria [37], handwriting images [38], credit scores [39], and remote sensing [40]. Here, we demonstrate that this approach is a significant improvement over alternative methods of machine learning-enabled leukocoria detection.

## Methods

### Ethics statement

This study was determined to be exempt from review by an Institutional Review Board at Baylor University. The parents of the study participants have given written informed consent to use and publish unaltered images of faces.

### Database and feature extraction

**Figure 2:**
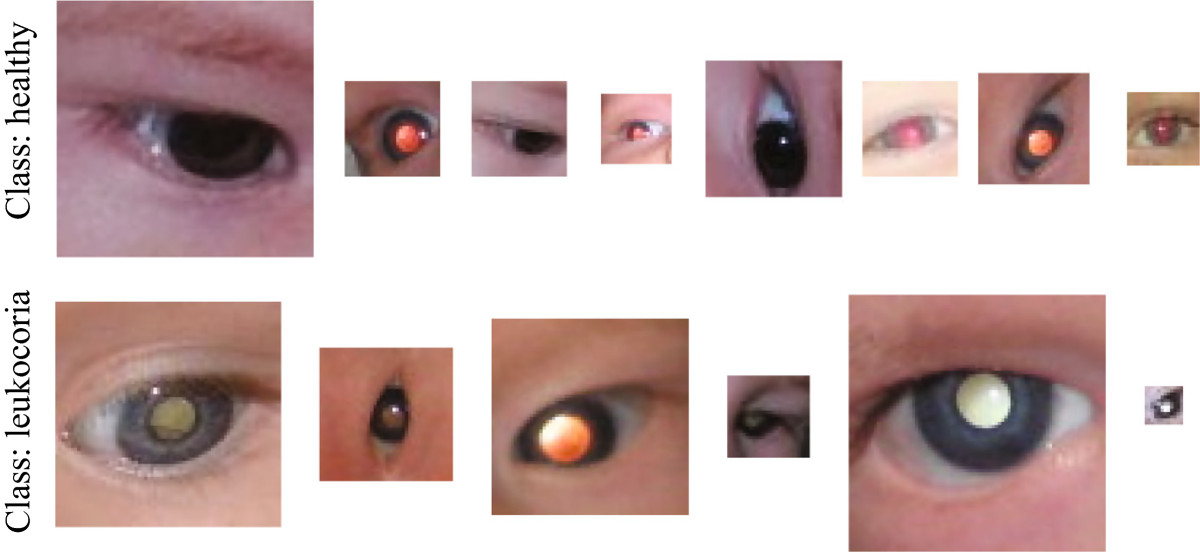
**Sample images from the experimental database.**

**Figure 3:**
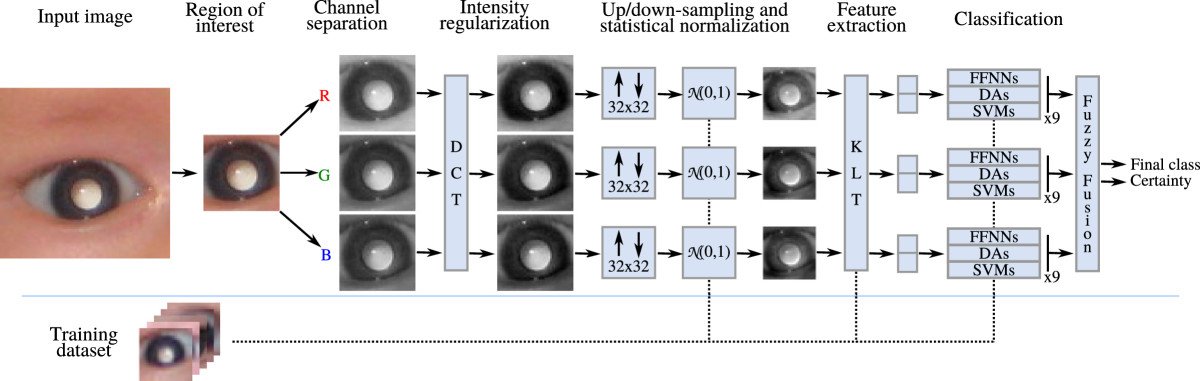
**Proposed image pre-processing strategy and feature extraction for the detection of leukocoria.**

This research uses a database of digital images corresponding to the eyes of 72 faces, for a total of 144 eye images. This database is strictly an internal collection of images produced by the authors of this paper, consequently, no external permission is required. To the best of our knowledge, there are no other databases for this task.Out of the 144 eye images, 54 eyes are labeled as "leukocoric" while the remaining 90 are labeled as "healthy". This implies that the database is unbalanced with 37.5% being the positive class and 62.5% the negative. The size of each image varies being 19 × 19 the smallest size and 138 × 138 the largest. Orientation, angle, and rotation of each eye varies from image to image. The database includes faces with different skin and iris color. Illumination is not controlled and varies depending of the distance between the face and the flash of the camera. Also, different cameras were used to build the database. Figure 2 depicts several example images from the database.Figure 2 shows samples for the two classes and illustrates the challenges mentioned above. These challenges demand a pre-processing strategy that reduces the effect of random factors in the acquisition process. We use the strategy explained herein and presented in Figure 3.

First, the input image is cropped to contain only the *M* × *N* image of the circumference delimited by the iris. This process can be done either manually or automatically.

Secondly, the cropped *M* × *N* three-channel (RGB) image, denoted as **I**(*n*_1_,*n*_2_,*n*_3_), where *n*_1_ ∈ {0,…,*M* - 1}, *n*_2_ ∈ {0,…,*N* - 1}, and *n*_3_ ∈ {0,1,2}, is separated into three different gray-scale images, **I**_*R*_(*n*_1_,*n*_2_), **I**_*G*_(*n*_1_,*n*_2_), and **I**_*B*_(*n*_1_,*n*_2_).

The next step leverages 2D-DCT to alleviate the problem of variant illumination in all three channels. For an image **I**(*n*_1_,*n*_2_) of size *M* × *N*, we can determine a matrix **F**_**I**_(*k*_1_,*k*_2_) also of size *M* × *N* that contains all the spatial frequency components of the image, for *k*_1_ ∈ {0,…,*M* - 1} and *k*_2_ ∈ {0,…,*N* - 1}. The matrix **F**_**I**_ can be computed with the 2D-DCT in the following manner:

1 $$\begin{array}{ll} \;F_{I}(k_{1},k_{2}) & =F\left\{I(n_{1},n_{2})\right\}\\ =\alpha (k_{1})\alpha (k_{2})\overset{M-1}{\underset{n_{1}=0}{\sum }}\overset{N-1}{\underset{n_{2}=0}{\sum }}I(n_{1},n_{2})\times \ldots \\ \;\cos\left[\frac{\pi }{M}\left(n_{1}+\frac{1}{2}\right)k_{1}\right]\times \ldots \\ \;\cos\left[\frac{\pi }{N}\left(n_{2}+\frac{1}{2}\right)k_{2}\right], \end{array}$$

where $F:R^{M\times N}\mapsto R^{M\times N}$ and

2 $$\begin{array}{l} \alpha (k)=\left\{\begin{array}{ll} \sqrt{\frac{1}{N}} & \text{for}\;k=0,\\ \sqrt{\frac{2}{N}} & \text{for}\;k\neq 0. \end{array}\right. \end{array}$$

According to [41], discarding the first three coefficients of **F**_**I**_(*k*_1_,*k*_2_) will counter the variation of illumination within the image. That is, an altered frequency domain matrix ${\hat{F}}_{I}(k_{1},k_{2})$ is created by discarding the elements in the coordinates (*k*_1_ = 0,*k*_2_ = 0), (*k*_1_ = 0,*k*_2_ = 1), and (*k*_1_ = 1,*k*_2_ = 0) of **F**_**I**_. After discarding the first three DCT coefficients, ${\hat{F}}_{I}$ is inversely transformed from the frequency domain to the spatial domain as follows:

3 $$\begin{array}{ll} \;\hat{I}(n_{1},n_{2}) & =F^{-1}\left\{{\hat{F}}_{I}(k_{1},k_{2})\right\}\\ =\overset{M-1}{\underset{k_{1}=0}{\sum }}\overset{N-1}{\underset{k_{2}=0}{\sum }}\alpha (k_{1})\alpha (k_{2}){\hat{F}}_{I}(k_{1},k_{2})\times \ldots \\ \;\cos\left[\frac{\pi }{M}\left(n_{1}+\frac{1}{2}\right)k_{1}\right]\times \ldots \\ \;\cos\left[\frac{\pi }{N}\left(n_{2}+\frac{1}{2}\right)k_{2}\right], \end{array}$$

where $F^{-1}:R^{M\times N}\mapsto R^{M\times N}$ and *α*(·) is also computed with (2).

Fourth, each image $\hat{I}$ is then down-sampled or up-sampled to a fixed size of 32 × 32. The selection of this particular size was determined experimentally, training several classifiers using different image sizes and choosing the size that produced the smallest classification error in the average case, which was 32 × 32. Note that this is a very small resolution compared to the natural resolution of recreational photographs.

Fifth, we z-score (subtract the mean and divide by the standard deviation) for each channel. The purpose is to have a dataset approximating a N(0,1) distribution at each channel. That is, having a dataset that follows a normal distribution with zero mean and unit variance at each channel. In order to determine the mean and standard deviation for z-scoring we only make use of all images available for training, *i.e.*, the training dataset. Images in the testing dataset will require the estimated mean and standard deviation estimated for the training dataset. We define $\tilde{I}$ as the image $\hat{I}$ that has been processed by up-sampling or down-sampling, subtraction of a mean image, and division by a standard deviation.

Finally, the Karhunen-Loeve Transform (KLT) is applied to the data using only the two eigenvectors whose corresponding eigenvalues are the largest of all [42, 43]. This procedure is analog to dimensionality reduction using Principal Component Analysis (PCA). Experimental research determined that the minimum number of eigenvectors that can be used without loss of generalization is two. We define **x**_*i*_ as a two-row vector defining the *i*-th eye image transformed using the KLT; that is, $x=T\{\tilde{I}\}$, where T{·} denotes the KLT. Therefore, the transformed training set per each individual channel is defined as $D={\left\{x_{i},d_{i}\right\}}_{i=1}^{N}$, where $x_{i}\in R^{2}$, *d*_*i*_ ∈ {-1,1} is the desired target class corresponding to the *i*-th vector (indicating normal or leukocoric), and *N* indicates the total number of training samples. Then, the training set  is used in the design of classifiers, which is explained in the next section.

### Classification architecture

The proposed classification scheme involves the fusion of different classifiers that are known to perform well individually. The purpose of the fusion is to achieve better performance than with individual classifiers [44]. The fusion of classifiers is also known as "combination of multiple classifiers" [45], "mixture of experts" [46], or "consensus aggregation" [47]. This paper uses fuzzy logic to combine different classifiers using the method proposed in [33, 34]. A fuzzy integral conceptualizes the idea of the method along with Sugeno’s *g*_*λ*_-fuzzy measure [48]. The different classifier performances define the importance that the fusion method will give to each classifier. We propose having nine different classifiers per channel, as shown in Figure 3. The total number of classifiers is 27. We perform the analysis of each channel aiming to observe which channel performs better and to determine its contribution to correct classification in further studies. A final class is given considering each classifier’s output at each channel. The following paragraphs explain the fusion methodology.

#### Soft fusion of classifiers

Revisiting [33] and [48] we have that a set function $g:2^{Y}\mapsto [0,1]$ is called a fuzzy measure if 1) *g*(0) = 0, g(Y)=1, 2) g(A)≤g(B) if A⊂B, and 3) if ${\left\{A_{i}\right\}}_{i=1}^{\infty }$ is an increasing sequence of measurable sets, then $\lim_{i\to \infty }g(A_{i})=g\left(\lim_{i\to \infty }A_{i}\right)$. This can be used to define the following equality:

4 $$\begin{array}{l} g(A\cup B)=g(A)+g(B)+\mathrm{\lambda g}(A)g(B), \end{array}$$

which is known as the *g*_*λ*_-fuzzy measure, for some *λ* > -1, all A,B⊂x, and A∩B=∅.

If we consider  as a finite set and h:Y↦[0,1] as a fuzzy subset of , then, the fuzzy integral over  of the function *h* w.r.t. a fuzzy measure *g* can be defined as follows:

5 $$\begin{array}{ll} \;h(y)\circ g(\cdot ) & =\underset{E\subseteq Y}{\text{max}}\left[\min\left(\underset{y\in E}{\text{min}}h(y),g(E)\right)\right]\\ =\underset{t\subseteq [0,1]}{\text{max}}\left[\min\left(t,g(C_{t})\right)\right], \end{array}$$

where $C_{t}=\left\{y|h(y)\geq t\right\}$. The equality in Equation 5 defines the agreement between the expectation and the evidence.

Particularly, let  define a finite set containing the outputs of *n* classifiers, that is, $Y=\{y_{1},y_{2},\ldots ,y_{n}\}$. Let h:Y↦[0,1] be a function that tells the certainty of a classifier’s output to belong to a given class (*i.e.* provides the "evidence"). Then, order the classifiers according to their current classification certainty, such that *h*(*y*_1_) ≥ *h*(*y*_2_) ≥ ⋯ ≥ *h*(*y*_*n*_). Then it follows to define the fuzzy integral *e* w.r.t. a fuzzy measure *g* over  as follows:

6 $$\begin{array}{l} e=\overset{n}{\underset{i=1}{\text{max}}}\left[\min\left(h(y_{i}),g(A_{i})\right)\right], \end{array}$$

where $A_{i}=\{y_{1},y_{2},\ldots ,y_{i}\}$. Furthermore, since *g* is a *g*_*λ*_-fuzzy measure, each value for $g(A_{i})$ can be computed using the following recursive equation:

7 $$\begin{array}{l} g(A_{i})=\left\{\begin{array}{ll} g(\{y_{1}\})=g^{1} & \text{for}\;i=1,\\ \begin{array}{l} g^{i}+g(A_{i-1})\\ +\lambda g^{i}g(A_{i-1}) \end{array} & \text{for}\;1<i\leq n, \end{array}\right. \end{array}$$

where *λ* is the unique root greater than -1 that can be obtained solving the following polynomial:

8 $$\begin{array}{l} \lambda +1=\overset{n}{\underset{i=1}{\prod }}\left(1+\lambda g^{i}\right), \end{array}$$

where *λ* ∈ (-1,+*∞*) and *λ* ≠ 0. However, in order to solve the polynomial, we need to estimate the densities *g*^*i*^ (*i.e.*, "the expectation"). The *i*-th density *g*^*i*^ defines the degree of importance the *i*-th classifier *y*_*i*_ has in the final classification. This densities can be estimated by an expert, or defined using a training dataset. In this research we defined the densities using the performance obtained from the data, and the process of experimentation will be explained later. In the following subsection we discuss briefly the classifiers used in this research.

#### Selection of classifiers

We are using three different kinds of classifiers: artificial neural network (ANN)-based, support vector machines (SVM)-based, and discriminant analysis (DA)-based. The three ANN-based classifiers we use for each channel have the same Feed-Forward (FF) architecture [49]; the difference lies in the number of neurons in each hidden layer. The two outputs of each neural network have softmax activation functions; the goal is to train the neural networks to approximate probability density functions of the problem and output the posterior probabilities at the output layer. Thus the output layer’s activation functions, softmax, act as the function *h* that maps the output of the classifier to values in the range [0,1] indicating classification certainty for either class. We used a partial subset of data and started training with three different groups: networks that randomly have between a) 2–5 neurons, b) 6–25 neurons, and c) 26–125 neurons. After a large number of experiments we concluded that the three best architectures were those shown in Table 1. The selection was performed based on those networks whose balanced error rate (BER) was the lowest in the average case.

**Table 1:** **Number of hidden neurons for each channel**

Channel	ANN_1_	ANN_2_	ANN_3_
Red	2	20	50
Green	3	10	15
Blue	2	3	5

*E.g.*, consider the third row of Table 1; for the blue channel, the best three architectures were those with two, three, and five neurons in the hidden layer; in contrast, the red channel exhibited the lowest errors using two, 20, and 50 neurons in the hidden layer. Intuitively, one can conclude that the training data for both green and blue channels is much simpler to classify than the data for the red channel.

Next, the SVM-based classifiers in this research are, by necessity, of the soft margin kind since the dataset has two non-linearly separable classes [50]. This research uses four SVMs; each has a different type of kernel function. The four SVM kernel functions are: 1) linear, 2) quadratic, 3) polynomial, and 4) radial basis function (RBF).

An SVM with linear kernel is the simplest form of a soft margin SVM; in practice it only performs a dot product, leaving the data in the input space. SVMs with a quadratic kernel are a particular case of a polynomial kernel of second degree. An RBF kernel is a preferred choice in research that offers little or no information about the dataset properties. SVMs can be very powerful, but its effectiveness, however, is tied up to an appropriate selection of its model parameters, a.k.a. hyper-parameters [51]. The traditional soft-margin SVM requires a hyper-parameter usually known as "regularization" parameter, *C*, that penalizes data-points incorrectly classified. Then, depending on the kernel choice, SVMs may have additional hyper-parameters; *e.g.*, the polynomial kernel requires a parameter *p* that defines the degree of the polynomial while the RBF kernel requires the parameter *τ* which controls the wideness in an exponential Gaussian-like function.

The typical method to find a "good" set of hyper-parameters is called "grid search", which some times can be computationally costly, especially if the data set is large. Thus, in order to accelerate the process of finding the hyper-parameters this research uses a quasi-optimal method to find the hyper-parameters based on optimization techniques [52]. The list of hyper-parameters used in our SVM-based classifiers appears in Table 2. The table shows the final values of *C*, *p*, and *τ* for each channel and the particular kernel choice. In the case of SVMs based on a polynomial kernel with a variable degree, it was found that a third degree polynomial produced better results; this is shown in the fourth column of Table 2.

**Table 2:** **Kernel choice and parameters used with SVMs**

	Kernel *K*(x_*i*_,x_*j*_) =			
	x_*i*_	${\left(x_{i}^{T}x_{j}+1\right)}^{p}$		$e^{-\frac{1}{2\tau^{2}}{\left||x_{i}-x_{j}|\right|}_{2}^{2}}$
Channel	Linear	Quad.	Poly.	RBF
		*p* **= 2**	*p* **= 3**	**(** *C* **,** *τ* **)**
Red	*C* = 7	*C* = 4	*C* = 0.5	(9, 0.5)
Green	*C* = 3	*C* = 2	*C* = 2	(33, 2)
Blue	*C* = 2	*C* = 1	*C* = 2	(0.13, 0.5)

The last choice of classifiers are based on discriminant analysis. Both Linear Discriminant Analysis (LDA) [53] and Quadratic Discriminant Analysis (QDA) [54] are closely related and are well known in the community for their simplicity and the robustness provided by statistical properties of the data. QDA and LDA achieve optimal results, in terms of probability theory, when the data in each class follows a Gaussian distribution independent and identically distributed (IID). Since this research uses the KLT, the data is close to being IID; however, the data is not actually IID, as in most real-life applications such as this research. LDA and QDA require no parameters except for the mean and covariance matrix estimates for each channel; these are computed from the training set . The experiments performed while training the classifiers and the soft fusion are discussed next.

### Experimental design

The soft fusion of *i* classifiers for detecting leukocoria requires an estimation of each classifier’s importance, *i.e.*, the *i*-th density *g*^*i*^. This research defined each classifier’s importance based on their individual performances using several different performance metrics and averaging the ranking in each individual metric. This section describes the experimental process of evaluating each classifier and the final value for *g*^*i*^ density corresponding to the *i*-th classifier.

#### Cross-validation

The whole database of eye images contains 144 examples. We divided the database into 10 groups of approximately equal size in order to use the well-known *K*-fold cross validation (CV) technique. Cross validation helps the researcher get an estimate of true classification performances [55]. This research uses 10-fold CV (*K* = 10) in order to determine the true importance of each classifier.

The database is divided in 10 groups of 14.4 data points in the average case. The methodology selects which points belong to each group randomly. Nine out of the 10 groups follow the pre-processing and feature extraction procedure explained earlier. Then the set of nine groups with its corresponding target classes *d*_*i*_ is defined as the training dataset $D={\left\{x_{i},d_{i}\right\}}_{i=1}^{N}$, where $x_{i}\in R^{2}$, *d*_*i*_ ∈ {-1,1}. Then, the 10th group (the one not used for training) is used as the testing set $K={\left\{x_{j},d_{j}\right\}}_{j=1}^{M}$, where *N* + *M* = 144. The process is repeated 10 times selecting a different combination of nine groups each time leaving the 10th out for testing. Finally, the performances obtained with each testing set are averaged. We ran 10-fold CV 100 times in order to have more meaningful results, averaging each instance of 100 CVs. This process reduces the uncertainty that the CV method will choose nearly the same sets of data for the 10 groups. The following paragraph explains the performance metrics used to rank the classifiers.

#### Performance metrics

Let us define the *i*-th difference *y*_*i*_ - *d*_*i*_ as the *i*-th "residual error", where *y*_*i*_ is the actual output of the classifier when the testing set input vector **x**_*i*_ is presented at its input, for all $\{x_{i},d_{i}\}\in K$. Commonly, machine learning researchers use the following statistical metrics to quantify performance based on the residual error: Root Mean Squared Error (RMSE) and Normalized Root Mean Squared Error (NRMSE). Such metrics are defined as follows:

9a $$\begin{array}{l} \text{RMSE}=\sqrt{\frac{1}{M}\overset{M}{\underset{i=1}{\sum }}{\left(y_{i}-d_{i}\right)}^{2}}, \end{array}$$

9b $$\begin{array}{l} \text{NRMSE}=\frac{1}{\sigma }\sqrt{\frac{1}{M}\overset{M}{\underset{i=1}{\sum }}{\left(y_{i}-d_{i}\right)}^{2}}, \end{array}$$

where *σ* is the standard deviation of *y*_*i*_.

From estimation theory it is known that if one has the residual error’s expected value equal to zero, and a unit variance, one may have achieved the least-squares solution to the problem, either linear or non-linear. Furthermore, it is understood that as the variance of the residual error approaches zero, the problem is better solved. Therefore, we want to measure both the expected value and the variance. Let us denote the expected value of the residual error *μ*_*ε*_ and the variance of the residual error $\sigma_{\varepsilon }^{2}=\text{Var}[y_{i}-d_{i}-\mu_{\varepsilon }]$ and their sample-based estimators as follows:

9c $$\begin{array}{l} \mu_{\varepsilon }=E\left[y_{i}-d_{i}\right]=\frac{1}{M}\overset{M}{\underset{i=1}{\sum }}\left(y_{i}-d_{i}\right) \end{array}$$

9d $$\begin{array}{l} \sigma_{\varepsilon }^{2}=E{\left[y_{i}-d_{i}-\mu_{\varepsilon }\right]}^{2}=\frac{1}{M-1}\overset{M}{\underset{i=1}{\sum }}{\left(y_{i}-d_{i}-\mu_{\varepsilon }\right)}^{2}, \end{array}$$

from where it is desired that both |*μ*_*ε*_|,*σ*_*ε*_ → 0 as *M* → *∞*.

On the other hand, some standard performance metrics for binary classification employ the well known *confusion matrix*. For binary classification, four possible prediction outcomes exist. A correct prediction is either a True Positive (*TP*) or a True Negative (*TN*), while an incorrect prediction is either a False Positive (*FP*) or a False Negative (*FN*). Here ‘Positive’ and ‘Negative’ correspond to the predicted label of the example.

From hereafter we denote *TP* as the total number of true positives, *TN* as the total number of true negatives, *FP* as the total number of false positives, and *FN* as the total number of false negatives in a classification event using a complete dataset, which in our case is the cross validation set . Such definitions allow us to use following performance metrics based on a confusion matrix:

9e $$\begin{array}{l} \text{Accuracy}=\text{ACC}=\frac{\text{TP}+\text{TN}}{\text{TP}+\text{FN}+\text{FP}+\text{TN}}, \end{array}$$

9f $$\begin{array}{l} \text{TP rate}=\text{TPR}=\frac{\text{TP}}{\text{TP}+\text{FN}}, \end{array}$$

9g $$\begin{array}{l} \text{FP rate}=\text{FPR}=\frac{\text{FP}}{\text{FP}+\text{TN}}, \end{array}$$

9h $$\begin{array}{l} \text{Specificity}=\text{SPC}=\frac{\text{TN}}{\text{FP}+\text{TN}}, \end{array}$$

9i $$\begin{array}{l} \text{Positive Predictive Value}=\text{PPV}=\frac{\text{TP}}{\text{TP}+\text{FP}}, \end{array}$$

9j $$\begin{array}{l} \text{Negative Predictive Value}=\text{NPV}=\frac{\text{TN}}{\text{TN}+\text{FN}}, \end{array}$$

9k $$\begin{array}{l} \text{False Discovery Rate}=\text{FDR}=\frac{\text{FP}}{\text{FP}+\text{TP}}, \end{array}$$

9l $$\begin{array}{l} \text{Matthews Correlation Coefficient}=\text{MCC}=\\ \frac{\text{TP}\times \text{TN}-\text{FP}\times \text{FN}}{\sqrt{\left(\text{TP}+\text{FP})(\text{TP}+\text{FN})(\text{TN}+\text{FP})(\text{TN}+\text{FN}\right)}}, \end{array}$$

9m $$\begin{array}{l} F_{1}\text{-Score}=2\times \frac{\left(\frac{\text{TP}}{\text{TP}+\text{FP}}\right)\times \left(\frac{\text{TP}}{\text{TP}+\text{FN}}\right)}{\left(\frac{\text{TP}}{\text{TP}+\text{FP}}\right)+\left(\frac{\text{TP}}{\text{TP}+\text{FN}}\right)}, \end{array}$$

9n $$\begin{array}{l} \text{Balanced Error Rate}=\text{BER}=\\ \frac{1}{2}\left(\frac{\text{FP}}{\text{TN}+\text{FP}}+\frac{\text{FN}}{\text{FN}+\text{TP}}\right). \end{array}$$

Note that in the literature, one might also find the above measures with different names; *e.g.*, TPR is also known as Sensitivity, SPC is also known as TN rate, PPV is also known as Precision, and the *F*_1_-Score is also known as the *F*-Measure.

In the literature, one can find other typical performance metric based on the area under Receiver Operating Characteristics (ROC) curve [56]. The area under the ROC curve, abbreviated AUC, provides a basis for judging whether a classifier performs realistically better than others in terms of the relationship between its TPR and FPR.

The last performance metric we use is the Cohen’s kappa measure *κ*. The *κ* measure scores the number of correct classifications independently for each class and aggregates them [57]. This way of scoring is less sensitive to randomness caused by a different number of examples in each class, therefore, it is less sensitive to class bias in training data.

All the performance measures described in Equations 9a through 9n need to be interpreted according to a desired outcome. Table 3 shows all the performance metrics discussed and their corresponding desired outcome; this will help interpret the results and rank the classifiers fairly well.

**Table 3:** **Performance metrics and their desired outcome**

Metric	Interval or domain	Desired
RMSE	$R^{+}$	The smallest value.
NRMSE	$R^{+}$	The smallest value.
|*μ*_*ε*_|	$R^{+}$	The smallest value.
*σ*_*ε*_	$R^{+}$	The smallest value.
ACC	[0,1]	One.
TPR	[0,1]	One.
FPR	[0,1]	Zero.
SPC	[0,1]	One.
PPV	[0,1]	One.
NPV	[0,1]	One.
FDR	[0,1]	Zero.
MCC	[-1,1]	One.
*F*_1_-Score	[0,1]	One.
BER	[0,1]	Zero.
AUC	[0,1]	One.
*κ*	[0,1]	One.

## Results

Tables 4, 5 and 6 show the average performance of each classifier over 100 experiments using different metrics. Each table ranks the classifiers on different color channel data: red, green, and blue, respectively. The number in parenthesis defines the rank of a classifier for that particular metric (in each row). A classifier ranked as "(1)" is the best among all the others, consequently, one ranked as "(9)" is the worst. The average rank of each classifier is shown in the last row of each table and this is used to determine the actual importance of each classifier. The *i*-th density, *g*^*i*^, is computed using the following expression:

10 $$\begin{array}{l} g^{i}=\frac{1}{r_{i}\Sigma_{r}}, \end{array}$$

where *r*_*i*_ is the average rank of each classifier and *Σ*_*r*_ is the sum of all classifier ranks. In this manner, the sum of all densities is equal to one, which is desired [33].

**Table 4:** **Rank of red channel classifiers by performance analysis**

	ANN_1_	ANN_2_	ANN_3_	DA_1_	DA_2_	SVM_1_	SVM_2_	SVM_3_	SVM_4_
RMSE	1.180 (8)	1.172 (7)	1.221 (9)	**1.097** (**1**)	1.146 (6)	1.103 (3)	1.144 (5)	1.124 (4)	1.100 (2)
NRMSE	1.214 (8)	1.206 (7)	1.257 (9)	**1.129** (**1**)	1.179 (6)	1.136 (3)	1.177 (5)	1.157 (4)	1.133 (2)
|*μ*_*ε*_|	0.136 (5)	0.041 (3)	**0.010** (**1**)	0.121 (4)	0.163 (7)	0.068 (2)	0.298 (9)	0.221 (8)	0.158 (6)
*σ*_*ε*_	1.171 (7)	1.173 (8)	1.223 (9)	1.094 (2)	1.138 (6)	1.105 (3)	1.108 (5)	1.106 (4)	**1.092** (**1**)
ACC	0.651 (8)	0.656 (7)	0.626 (9)	**0.699** (**1**)	0.672 (6)	0.696 (3)	0.673 (5)	0.684 (4)	0.697 (2)
TPR	**0.775** (**1**)	0.741 (2)	0.697 (5)	0.711 (4)	0.672 (7)	0.729 (3)	0.619 (9)	0.659 (8)	0.694 (6)
FPR	0.556 (9)	0.486 (7)	0.492 (8)	0.320 (4)	0.329 (5)	0.360 (6)	**0.238** (**1**)	0.274 (2)	0.298 (3)
SPC	0.444 (9)	0.514 (7)	0.508 (8)	0.680 (4)	0.671 (5)	0.640 (6)	**0.762** (**1**)	0.726 (2)	0.702 (3)
PPV	0.700 (9)	0.718 (7)	0.703 (8)	0.787 (4)	0.773 (5)	0.771 (6)	**0.813** (**1**)	0.800 (2)	0.795 (3)
NPV	0.545 (7)	0.544 (8)	0.502 (9)	0.585 (2)	0.551 (5)	**0.586** (**1**)	0.545 (6)	0.560 (4)	0.579 (3)
FDR	0.300 (9)	0.282 (7)	0.297 (8)	0.213 (4)	0.227 (5)	0.229 (6)	**0.187** (**1**)	0.200 (2)	0.205 (3)
MCC	0.232 (8)	0.259 (7)	0.206 (9)	0.381 (2)	0.333 (6)	0.363 (5)	0.370 (4)	0.372 (3)	**0.385** (**1**)
*F*_1_	0.735 (4)	0.729 (5)	0.699 (9)	0.747 (2)	0.719 (7)	**0.750** (**1**)	0.703 (8)	0.722 (6)	0.741 (3)
BER	0.390 (8)	0.372 (7)	0.397 (9)	0.305 (2)	0.329 (6)	0.316 (5)	0.309 (4)	0.308 (3)	**0.302** (**1**)
AUC	0.610 (8)	0.628 (7)	0.603 (9)	0.695 (2)	0.671 (6)	0.684 (5)	0.691 (4)	0.692 (3)	**0.698** (**1**)
*κ*	0.228 (8)	0.258 (7)	0.205 (9)	0.378 (2)	0.329 (6)	0.362 (4)	0.353 (5)	0.363 (3)	**0.380** (**1**)
Avg.	7.29	6.47	8.06	**2.47**	5.88	**3.82**	4.59	3.88	**2.53**

The data in boldface indicates the best ranked method of each row, with the exception of the last row, which indicates the best three classifiers.

**Table 5:** **Rank of green channel classifiers by performance analysis**

	ANN_1_	ANN_2_	ANN_3_	DA_1_	DA_2_	SVM_1_	SVM_2_	SVM_3_	SVM_4_
RMSE	0.787 (4)	0.791 (5)	0.800 (7)	0.780 (3)	0.828 (8)	0.796 (6)	0.838 (9)	0.706 (2)	**0.673** (**1**)
NRMSE	0.810 (4)	0.814 (5)	0.823 (7)	0.802 (3)	0.853 (8)	0.819 (6)	0.863 (9)	0.727 (2)	**0.693** (**1**)
|*μ*_*ε*_|	0.030 (3)	0.025 (2)	**0.028** (**1**)	0.075 (5)	0.059 (4)	0.107 (8)	0.137 (9)	0.081 (7)	0.078 (6)
*σ*_*ε*_	0.788 (4)	0.792 (6)	0.801 (7)	0.779 (3)	0.829 (8)	0.791 (5)	0.830 (9)	0.704 (2)	**0.671** (**1**)
ACC	0.845 (4)	0.843 (5)	0.839 (7)	0.848 (3)	0.828 (8)	0.842 (6)	0.824 (9)	0.875 (2)	**0.887** (**1**)
TPR	**0.888** (**1**)	0.884 (2)	0.883 (3)	0.848 (6)	0.839 (7)	0.831 (8)	0.805 (9)	0.868 (5)	0.878 (4)
FPR	0.227 (8)	0.226 (7)	0.233 (9)	0.153 (5)	0.189 (6)	0.140 (3)	0.143 (4)	0.113 (2)	**0.099** (**1**)
SPC	0.773 (8)	0.774 (7)	0.767 (9)	0.847 (5)	0.811 (6)	0.860 (3)	0.857 (4)	0.887 (2)	**0.901** (**1**)
PPV	0.867 (8)	0.868 (7)	0.864 (9)	0.903 (5)	0.881 (6)	0.908 (3)	0.904 (4)	0.928 (2)	**0.937** (**1**)
NPV	0.806 (2)	0.802 (3)	0.797 (5)	0.770 (6)	0.751 (8)	0.753 (7)	0.725 (9)	0.801 (4)	**0.816** (**1**)
FDR	0.133 (8)	0.132 (7)	0.136 (9)	0.097 (5)	0.119 (6)	0.092 (3)	0.096 (4)	0.072 (2)	**0.063** (**1**)
MCC	0.667 (5)	0.664 (6)	0.656 (7)	0.684 (3)	0.641 (9)	0.676 (4)	0.645 (8)	0.742 (2)	**0.766** (**1**)
*F*_1_	0.877 (3)	0.876 (4)	0.873 (6)	0.875 (5)	0.859 (8)	0.868 (7)	0.851 (9)	0.897 (2)	**0.906** (**1**)
BER	0.170 (6)	0.171 (7)	0.175 (8)	0.152 (3)	0.175 (9)	0.155 (4)	0.169 (5)	0.123 (2)	**0.110** (**1**)
AUC	0.830 (6)	0.829 (7)	0.825 (8)	0.848 (3)	0.825 (9)	0.845 (4)	0.831 (5)	0.877 (2)	**0.890** (**1**)
*κ*	0.666 (5)	0.663 (6)	0.655 (7)	0.682 (3)	0.639 (8)	0.672 (4)	0.638 (9)	0.739 (2)	**0.763** (**1**)
Avg.	4.88	5.35	6.82	**4.06**	7.41	5.12	7.29	**2.59**	**1.47**

The data in boldface indicates the best ranked method of each row, with the exception of the last row, which indicates the best three classifiers.

**Table 6:** **Rank of blue channel classifiers by performance analysis**

	ANN_1_	ANN_2_	ANN_3_	DA_1_	DA_2_	SVM_1_	SVM_2_	SVM_3_	SVM_4_
RMSE	0.863 (8)	0.858 (7)	0.851 (6)	0.827 (4)	0.866 (9)	0.803 (3)	0.848 (5)	**0.791** (**1**)	0.792 (2)
NRMSE	0.888 (8)	0.883 (7)	0.876 (6)	0.851 (4)	0.891 (9)	0.826 (3)	0.873 (5)	**0.814** (**1**)	0.815 (2)
|*μ*_*ε*_|	0.063 (9)	0.058 (7)	0.063 (8)	0.024 (2)	0.029 (4)	0.043 (6)	0.029 (3)	**0.018** (**1**)	0.036 (5)
*σ*_*ε*_	0.862 (8)	0.858 (7)	0.851 (6)	0.830 (4)	0.868 (9)	0.805 (3)	0.851 (5)	**0.793** (**1**)	0.794 (2)
ACC	0.813 (8)	0.816 (7)	0.818 (6)	0.829 (4)	0.813 (9)	0.839 (3)	0.820 (5)	**0.844** (**1**)	0.843 (2)
TPR	0.876 (2)	0.876 (3)	**0.880** (**1**)	0.853 (7)	0.838 (9)	0.854 (6)	0.844 (8)	0.868 (4)	0.860 (5)
FPR	0.291 (9)	0.284 (8)	0.284 (7)	0.212 (4)	0.230 (6)	0.186 (2)	0.221 (5)	0.197 (3)	**0.186** (**1**)
SPC	0.709 (9)	0.716 (8)	0.716 (7)	0.788 (4)	0.770 (6)	0.814 (2)	0.779 (5)	0.803 (3)	**0.814** (**1**)
PPV	0.834 (9)	0.838 (8)	0.838 (7)	0.870 (4)	0.858 (6)	0.884 (2)	0.864 (5)	0.880 (3)	**0.885** (**1**)
NPV	0.775 (5)	0.776 (4)	0.782 (2)	0.763 (7)	0.741 (9)	0.770 (6)	0.750 (8)	**0.784** (**1**)	0.777 (3)
FDR	0.166 (9)	0.162 (8)	0.162 (7)	0.130 (4)	0.142 (6)	0.116 (2)	0.136 (5)	0.120 (3)	**0.115** (**1**)
MCC	0.597 (9)	0.602 (8)	0.608 (6)	0.638 (4)	0.604 (7)	0.661 (3)	0.619 (5)	0.668 (2)	**0.669** (**1**)
*F*_1_	0.854 (8)	0.856 (6)	0.858 (5)	0.862 (4)	0.848 (9)	0.869 (3)	0.854 (7)	**0.874** (**1**)	0.873 (2)
BER	0.208 (9)	0.204 (8)	0.202 (7)	0.179 (4)	0.196 (6)	0.166 (3)	0.188 (5)	0.165 (2)	**0.163** (**1**)
AUC	0.792 (9)	0.796 (8)	0.798 (7)	0.821 (4)	0.804 (6)	0.834 (3)	0.812 (5)	0.835 (2)	**0.837** (**1**)
*κ*	0.595 (9)	0.600 (8)	0.606 (6)	0.637 (4)	0.603 (7)	0.660 (3)	0.619 (5)	0.668 (2)	**0.668** (**1**)
Avg.	8.00	7.00	5.88	4.24	7.41	**3.29**	5.35	**1.88**	**1.94**

The data in boldface indicates the best ranked method of each row, with the exception of the last row, which indicates the best three classifiers.

From Table 4 we observe that for the red channel, the first three best ranked classifiers are LDA (DA _1_), and SVM with RBF kernel (SVM _4_), and SVM linear (SVM _1_). Table 5 shows that for the green channel, SVM with RBF kernel, SVM with polynomial kernel of third degree (SVM _3_), and LDA as the best ranked classifiers respectively. Similarly, Table 6 shows that for the blue channel, the SVM with polynomial kernel of third degree, SVM with RBF kernel, and SVM linear are the top three classifiers respectively.

### Soft fusion classification and comparison

Finally, we can perform the soft fusion of classifiers using the densities found after performance analysis of the classifiers. Since the densities, *g*^*i*^, are now known, we can use Equation 8 to determine the appropriate value for *λ* and then compute the *g*_*λ*_-fuzzy measure using Equation 7 that allows us to compute the fuzzy integral (Equation 6).

For comparison purposes we also use three of the most common combination methods: 1) Average, 2) Weighted Average, and 3) Majority. The Average method consists of averaging the classification of all classifiers and choosing the class closest to the average. However, the Weighted Average method takes into account the importance of each classifier as determined by the densities *g*^*i*^ and multiplies each classifier’s output by its corresponding importance; the products are added all together and the method decides for the class closest to the sum. In contrast, the majority method considers all classifiers equally relevant and takes a vote, deciding for class that agrees with the majority. Note that the Average and Majority methods produce the value for metrics based on classification error (such as Accuracy and TPR), but differ in metrics producing real values (such as RMSE). This is because the Average method uses real values output from the individual models, while the Majority method uses voting.

Table 7 shows the results of classification with the different methods of combining classifiers. Note that these methods consider the information of all classifiers in all three channels and, thus, only one table is necessary. The next section introduces the analysis of these results. Note, however, that in the next section, the variables *p* and *α* are redefined and have the traditional meaning of statistical analysis and they shall not be confused with the variables *p* and *α* that, in the rest of the paper, represent a kernel parameter and a DCT scaling function, respectively.

**Table 7:** **Performance analysis of different methods of classifier combination**

	Average	Weighted avg.	Majority	Soft fusion
RMSE	0.682 ± 0.021(3)	0.674 ± 0.021(2)	0.705 ± 0.030(4)	**0.652 ± 0.014(1)**
NRMSE	0.702 ± 0.021(3)	0.694 ± 0.021(2)	0.725 ± 0.031(4)	**0.671 ± 0.014(1)**
|*μ*_*ε*_|	**0.058 ± 0.018(1)**	0.065 ± 0.016(2)	0.071 ± 0.023(3)	0.114 ± 0.008(4)
*σ*_*ε*_	0.682 ± 0.021(3)	0.673 ± 0.021(2)	0.703 ± 0.032(4)	**0.644 ± 0.015(1)**
ACC	0.876 ± 0.011(3)	0.876 ± 0.011(3)	0.876 ± 0.011(3)	**0.881 ± 0.011(1)**
TPR	0.872 ± 0.009(3)	0.872 ± 0.009(3)	0.872 ± 0.009(3)	**0.878 ± 0.008(1)**
FPR	0.119 ± 0.026(3)	0.119 ± 0.026(3)	0.119 ± 0.026(3)	**0.114 ± 0.024(1)**
SPC	0.881 ± 0.026(3)	0.881 ± 0.026(3)	0.881 ± 0.026(3)	**0.886 ± 0.024(1)**
PPV	0.925 ± 0.015(3)	0.925 ± 0.015(3)	0.925 ± 0.015(3)	**0.928 ± 0.014(1)**
NPV	0.805 ± 0.011(3)	0.805 ± 0.011(3)	0.805 ± 0.011(3)	**0.813 ± 0.011(1)**
FDR	0.075 ± 0.015(3)	0.075 ± 0.015(3)	0.075 ± 0.015(3)	**0.072 ± 0.014(1)**
MCC	0.742 ± 0.024(3)	0.742 ± 0.024(3)	0.742 ± 0.024(3)	**0.752 ± 0.023(1)**
*F*_1_	0.898 ± 0.008(3)	0.898 ± 0.008(3)	0.898 ± 0.008(3)	**0.902 ± 0.008(1)**
BER	0.123 ± 0.013(3)	0.123 ± 0.013(3)	0.123 ± 0.013(3)	**0.118 ± 0.013(1)**
AUC	0.891 ± 0.009(3)	0.891 ± 0.009(2)	0.877 ± 0.013(4)	**0.918 ± 0.007(1)**
*κ*	0.739 ± 0.024(3)	0.739 ± 0.024(3)	0.739 ± 0.024(3)	**0.750 ± 0.023(1)**
Avg. SD	0.0169	0.0168	0.0196	**0.0141**
Avg. Rank	2.8824	2.6471	3.1176	**1.3529**

The data in boldface indicates the best ranked classification method of each row.

## Discussion

Table 7 shows that the proposed classification scheme performs better than the other three methodologies in most cases. The soft fusion of classifiers produces results that have less variability in the average case, as shown in the second-to-last row.

The results in Tables 4, 5 and 6 clearly indicate that classifiers that use the green channel information perform better than those using blue or red channel information. Also, we can observe that the classifiers using red channel information perform the worst of all. Therefore, we can argue that the most discriminant information is carried over the green channel and the information in the red channel may be introducing noise to the soft fusion of classifiers. Considering this possibility we compare the results of the best classifiers that use the information of the green channel against the proposed scheme, *i.e.*, SVM with RBF kernel from Table 5 against the soft fusion method in Table 7. In comparison we can notice that the proposed soft fusion of classifiers performs better only in terms of the RMSE, NRMSE, *σ*_*ε*_, and AUC. This means that the proposed scheme has better statistical stability, and that its relationship in terms of TPR and FPR demonstrates better performance. In all the remaining instances the SVM classifier with RBF kernel performs better than the soft fusion; arguably, because of the introduction of noise via red channel information.

We continued by performing the well known Friedman’s test and if the null-hypothesis were rejected we also performed the post-hoc Nemenyi’s test [58]. First, Friedman’s test determined that the results were statistically significant with *p* = 1.12 × 10^-5^ rejecting the null-hypothesis. The null-hypothesis being tested here is that the different approaches presented in the comparison of Table 7 perform the same, and that their performance differences are random. Then, since the null hypothesis was rejected it followed to perform the post hoc Nemenyi’s test. We determined the critical difference (CD) for comparing four methods of combining classifiers using 17 different performance metrics with a level of significance *α* = 0.05. The result is the following: $\text{CD}=2.569\sqrt{\frac{4\times 5}{6\times 17}}=1.1376$. Therefore, since the difference between the two best methods, *i.e.*, Weighted Average and Soft Fusion, is greater than the CD, then we conclude that the Soft Fusion of classifiers performs significantly better than the other three methods in a statistical sense. That is, 2.6471 - 1.3529 = 1.2942 > 1.1376. Note that even when both the Soft Fusion and Weighted Average methods take the importance of each classifier into account, still the proposed classification scheme is significantly better.

**Figure 4:**
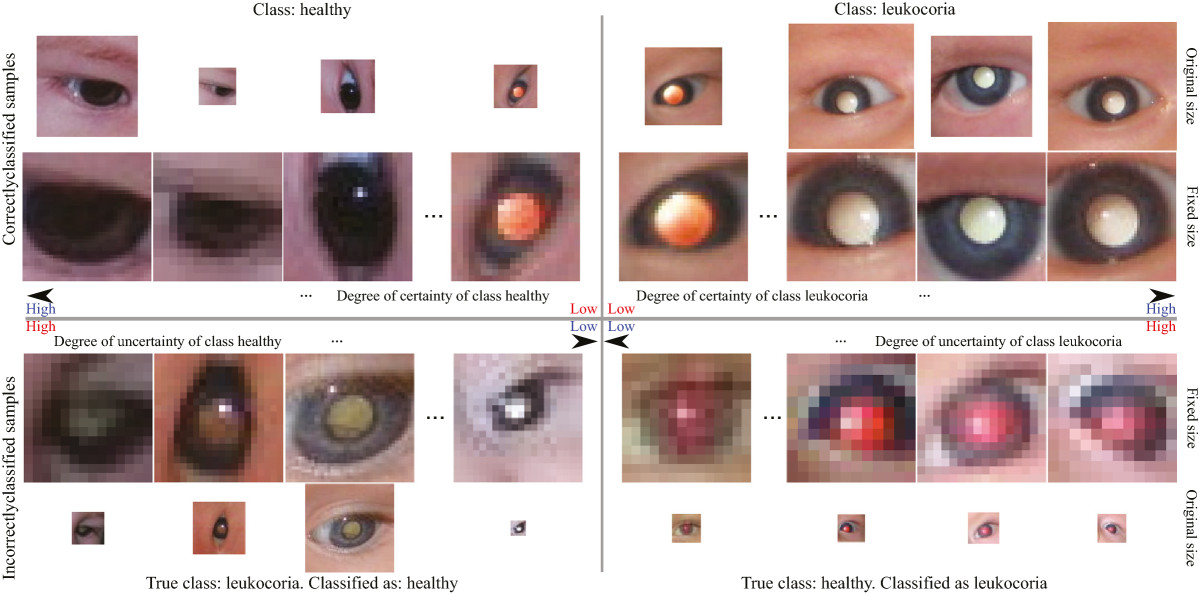
**Analysis of classification certainty and uncertainty as the eye images are classified as healthy or leukocoric.**

Figure 4 depicts an analysis of the classification certainty and uncertainty. This analysis is possible since the fuzzy integral (Equation 6) gives us the certainty that a classifier’s ouptut *y*_*i*_ belongs to one class or the other. From the upper part of Figure 4 we can observe how images in the threshold of being misclassified as leukocoric or misclassified as healthy are extremely similar and, thus, difficult to classify. The lower part of Figure 4 illustrates the problem when images are in the threshold of being correctly classified as healthy or leukocoric; here the problem seems to be related to the resolution of the original image. The lower the resolution the higher the risk of the image to be misclassified. Also the angle towards where the eye is gazing affects the classification to some degree. This is expected since the white reflection of the leukocoric eye is better observed when the eye is looking directly towards the camera and its source of light; the converse is also true and affects classification. Skin color and uneven illumination problems were reduced because of the image preprocessing explained earlier; however, experimental proof of this remains pending for further publications.

## Conclusions

The proposed classification scheme presented in this research uses a soft fusion of multichannel classifiers that are experts in detecting leukocoria in human eyes. These experts are trained with features extracted from RGB images preprocessed to overcome poor illumination and skin color variation using the DCT, statistical normalization of the images, and the KLT.

This research uses nine different classifiers per channel for a total of 27 experts. These include neural networks, linear discriminant classifiers, and support vector machines. The estimation of the fuzzy densities, a.k.a. importance of classifiers, was determined experimentally using cross-validation. The null-hypothesis was rejected and we demonstrated that the proposed classification scheme performs significantly better than the other approaches. Furthermore, it was shown that the green channel provides with more discriminant information than the other two.

While a soft fusion of classifiers is a good alternative in the detection of leukocoria in eyes of infants, it is just one part of a larger program to identify leukocoria in natural images. Other areas of research include eye localization (to improve detection), age discrimination (to reduce false positives on adult subjects), and alternative learning-based methods for leukocoria detection [59, 60].

## Consent

Written informed consent was obtained from the patient’s parents for the publication of this report and any accompanying images.

## Authors’ original submitted files for images

Below are the links to the authors’ original submitted files for images. Authors’ original file for figure 1 Authors’ original file for figure 2 Authors’ original file for figure 3 Authors’ original file for figure 4
