# Supplementary material for: Detection of leukocoria using a soft fusion of expert classifiers under non-clinical settings
Source: BMC Ophthalmol. 2014 Sep 9;14:110. doi: 10.1186/1471-2415-14-110 (PMC4167153; doi:10.1186/1471-2415-14-110)
Supplement: Supplementary file 1 — Authors’ original file for figure 1 [file 12886_2014_472_MOESM1_ESM.pdf]

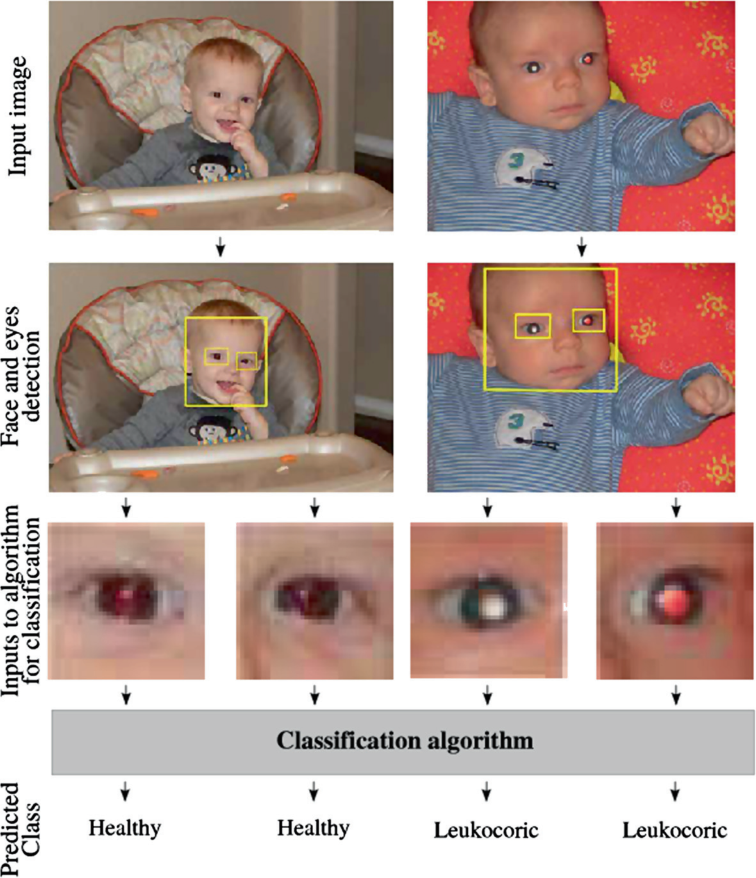
Input image
Face and eyes detection
Inputs to algorithm for classification
Classification algorithm
Predicted Class
Healthy
Healthy
Leukocoric
Leukocoric
