# Supplementary figures and images for: Detection of leukocoria using a soft fusion of expert classifiers under non-clinical settings
Source: BMC Ophthalmol. 2014 Sep 9;14:110. doi: 10.1186/1471-2415-14-110 (PMC4167153; doi:10.1186/1471-2415-14-110)

Class: leukocoria

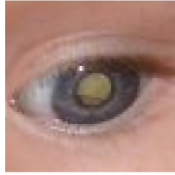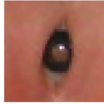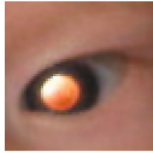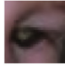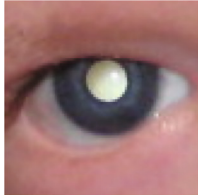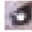

Class: healthy

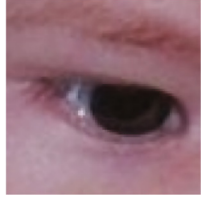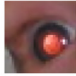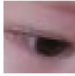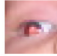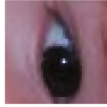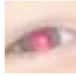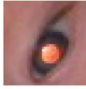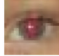

Supplement: Supplementary file 2 — Authors’ original file for figure 2 [file 12886_2014_472_MOESM2_ESM.pdf]

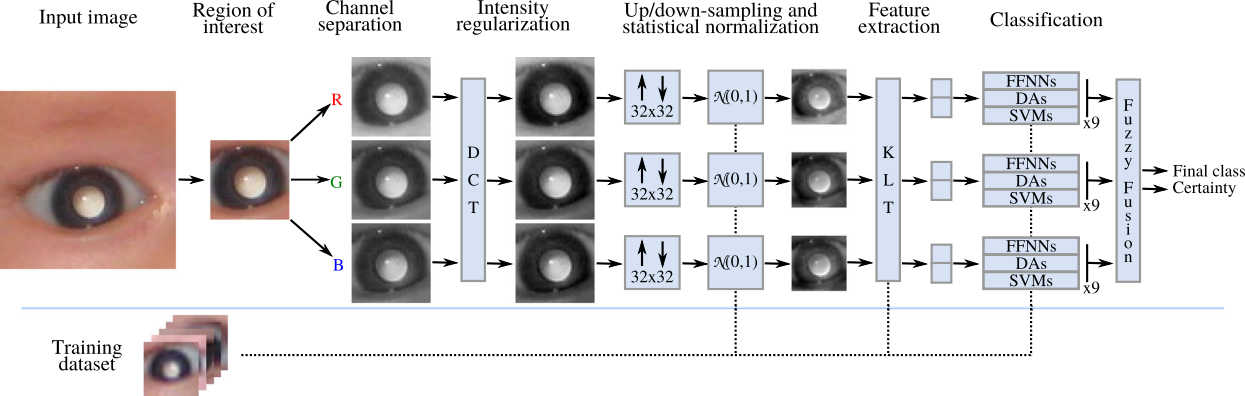

Supplement: Supplementary file 3 — Authors’ original file for figure 3 [file 12886_2014_472_MOESM3_ESM.pdf]
