# Supplementary material for: Detection of leukocoria using a soft fusion of expert classifiers under non-clinical settings
Source: BMC Ophthalmol. 2014 Sep 9;14:110. doi: 10.1186/1471-2415-14-110 (PMC4167153; doi:10.1186/1471-2415-14-110)
Supplement: Supplementary file 4 — Authors’ original file for figure 4 [file 12886_2014_472_MOESM4_ESM.pdf]

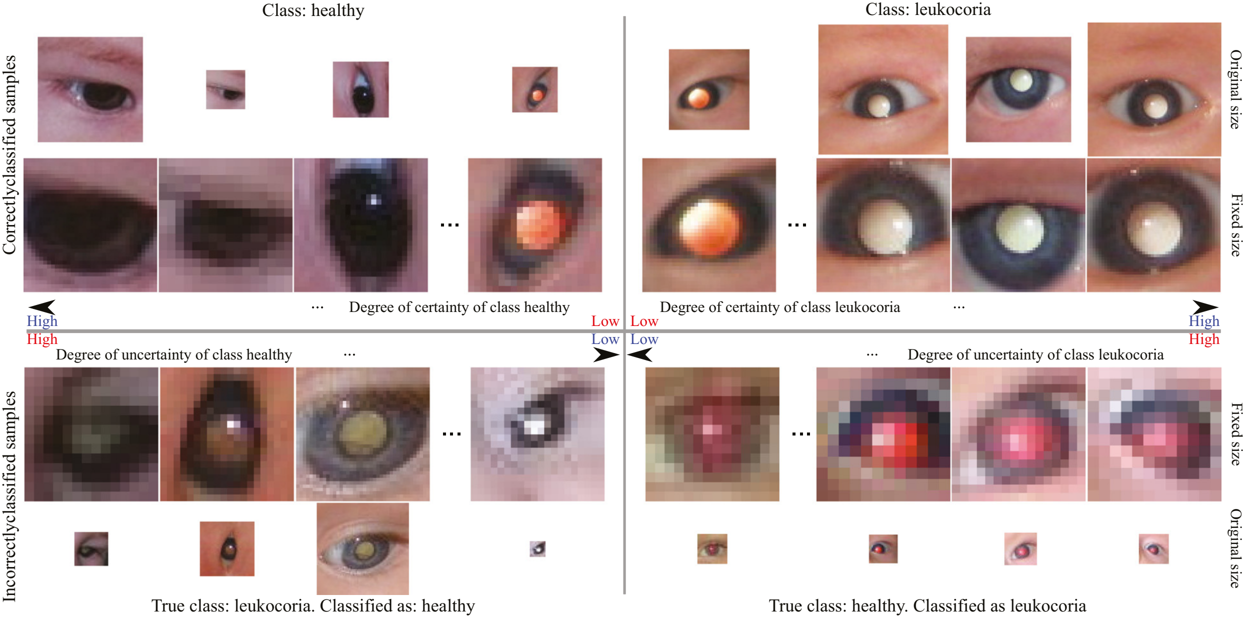
Class: healthy
Class: leukocoria
Correctlyclassified samples
Original size
Fixed size
Degree of certainty of class healthy
Degree of certainty of class leukocoria
High
Low
Low
High
High
Low
Low
High
Degree of uncertainty of class healthy
Degree of uncertainty of class leukocoria
Incorrectlyclassified samples
Fixed size
Original size
True class: leukocoria. Classified as: healthy
True class: healthy. Classified as leukocoria
